# The Plasmodium falciparum Cell-Traversal Protein for Ookinetes and Sporozoites as a Candidate for Preerythrocytic and Transmission-Blocking Vaccines

**DOI:** 10.1128/IAI.00498-16

**Published:** 2017-01-26

**Authors:** Diego A. Espinosa, Joel Vega-Rodriguez, Yevel Flores-Garcia, Amy R. Noe, Christian Muñoz, Russell Coleman, Torben Bruck, Keith Haney, Alex Stevens, Diane Retallack, Jeff Allen, Thomas S. Vedvick, Christopher B. Fox, Steven G. Reed, Randall F. Howard, Ahmed M. Salman, Chris J. Janse, Shahid M. Khan, Fidel Zavala, Gabriel M. Gutierrez

**Affiliations:** a Department of Molecular Microbiology and Immunology, Malaria Research Institute, Johns Hopkins Bloomberg School of Public Health, Baltimore, Maryland, USA; b Leidos Life Sciences, Frederick, Maryland, USA; c Pfenex Inc., San Diego, California, USA; d Infectious Disease Research Institute, Seattle, Washington, USA; e The Jenner Institute, Nuffield Department of Medicine, University of Oxford, Oxford, United Kingdom; f Leiden Malaria Research Group, Department of Parasitology, Center of Infectious Diseases, Leiden University Medical Center, Leiden, The Netherlands; University of South Florida

**Keywords:** CelTOS, parasite, malaria, transgenic, vaccine

## Abstract

Recent studies have shown that immune responses against the cell-traversal protein for Plasmodium ookinetes and sporozoites (CelTOS) can inhibit parasite infection. While these studies provide important evidence toward the development of vaccines targeting this protein, it remains unknown whether these responses could engage the Plasmodium falciparum CelTOS *in vivo*. Using a newly developed rodent malaria chimeric parasite expressing the P. falciparum CelTOS (PfCelTOS), we evaluated the protective effect of *in vivo* immune responses elicited by vaccination and assessed the neutralizing capacity of monoclonal antibodies specific against PfCelTOS. Mice immunized with recombinant P. falciparum CelTOS in combination with the glucopyranosyl lipid adjuvant-stable emulsion (GLA-SE) or glucopyranosyl lipid adjuvant-liposome-QS21 (GLA-LSQ) adjuvant system significantly inhibited sporozoite hepatocyte infection. Notably, monoclonal antibodies against PfCelTOS strongly inhibited oocyst development of P. falciparum and Plasmodium berghei expressing PfCelTOS in Anopheles gambiae mosquitoes. Taken together, our results demonstrate that anti-CelTOS responses elicited by vaccination or passive immunization can inhibit sporozoite and ookinete infection and impair vector transmission.

## INTRODUCTION

The most recent report by the World Health Organization indicates that in 2013 there were approximately 198 million malaria cases worldwide and an estimated 584,000 deaths (1). While global efforts to reduce the impact of the disease have shown progress over the last decade, malaria still represents a major burden to humanity, especially to populations living in African countries. Strategic vector-control interventions, accurate diagnostic tests, and effective antimalarial drugs are fundamental requirements in the fight against malaria. However, it is acknowledged that the development of a fully efficient vaccine is critical to achieve malaria eradication.

To date, RTS,S is the most advanced malaria vaccine candidate. This formulation is based on a portion of the most abundant protein on the surface of the malaria sporozoite, the circumsporozoite protein (CSP). Notably, RTS,S has shown potential to decrease the incidence of severe malaria in children and to prevent infection in 30 to 50% of its recipients (2–4). While these findings are encouraging, additional improvements may be required to accomplish a more broadly protective formulation.

A likely way to increase the effectiveness of subunit malaria vaccines is the development of formulations incorporating multiple parasite antigens (5). Besides CSP, a number of recent studies have explored the protective effect of immune responses targeting different sporozoite antigens (6–9). One of these antigens is the cell-traversal protein for ookinetes and sporozoites (CelTOS), which has a critical role in the establishment of malaria infections in both mosquito and vertebrate hosts (10). Importantly, antibody and T-cell responses against CelTOS have shown to mediate cross-species protection against a heterologous Plasmodium species challenge and to confer sterile protection to immunized mice (11).

In this study, we utilized the Pfenex platform to express Plasmodium falciparum CelTOS (PfCelTOS) protein that can be grown and purified under good manufacturing practices. Further, we report on the use of a newly generated chimeric Plasmodium berghei parasite expressing the PfCelTOS and demonstrate that immune responses against PfCelTOS can inhibit sporozoite hepatocyte infection. In addition, we also show that monoclonal antibodies against this protein inhibit sporozoite infectivity *in vivo* and significantly impair parasite development in Anopheles gambiae mosquitoes. Our results demonstrate that immune responses against CelTOS not only have the potential to inhibit Plasmodium infection but also decrease malaria transmission.

## RESULTS

### Expression and purification of recombinant CelTOS protein.

Plasmodium falciparum CelTOS (PfCelTOS) protein has previously been produced cytoplasmically in Escherichia coli (11); however, this material was generated on a small scale using an affinity tag, adding 16 amino acids to the N terminus of mature PfCelTOS. To enable vaccine development and later clinical manufacturing, a Pseudomonas fluorescens production strain encoding mature CelTOS with no added amino acids was developed. A gene encoding mature CelTOS, optimized for expression in P. fluorescens, by DNA2.0 using DNA2.0's proprietary algorithm in combination with the codon usage table for Pfenex's P. fluorescens strain MB214, was fused in frame with a variety of secretion leaders (12) to generate expression plasmids, which were screened in combination with an array of host strains to identify an optimal production strain. Samples grown at a 0.5-ml scale were evaluated for titer and intact mass. Strains with low levels of clipping (<0.3%) that showed titers of up to 0.2 g/liter at the 0.5-ml scale were selected to advance to 2-liter bioreactors to produce material for preclinical testing. Unoptimized titers ranged from ∼0.7 to over 1 g/liter. A three-step purification scheme was developed to purify recombinant PfCelTOS (PfrCelTOS). The final purified material had low endotoxin levels (<12 endotoxin units [EU]/mg) and corresponded to fully intact mature CelTOS, with no detectable clipped species.

### Generation of PbANKA-PfCelTOS(r)_PbCelTOS_CelTOS chimeric parasites.

We developed a rodent challenge model, which involved creating a chimeric P. berghei parasite line where the P. berghei *celtos* coding sequence (CDS) (Pb*celtos*) gene was replaced with the P. falciparum *celtos* CDS (Pf*celtos*), amplified from genomic DNA of the P. falciparum NF54 strain. In addition to expressing PfCelTOS, these chimeric parasites constitutively express the fusion green fluorescent protein (GFP)-luciferase reporter protein (see Fig. S1 and S3 in the supplemental material). Correct replacement of the Pb*celtos* CDS by the Pf*celtos* CDS in the chimeric line was confirmed by diagnostic Southern analysis of chromosomes separated by pulsed-field gel electrophoresis and diagnostic PCR on genomic DNA (Fig. S1). Immunofluorescence microscopy of chimeric and wild-type (WT) sporozoites using sera from mice immunized with PfCelTOS and PbCelTOS (13) confirmed the expression of PfCelTOS in sporozoites of chimeric parasite line 2258cl2 (Fig. S1). Chimeric parasites showed normal asexual blood stage multiplication in mice (data not shown), and oocyst and sporozoite production in Anopheles stephensi mosquitoes was comparable to that of WT P. berghei parasites (see Table S3 in the supplemental material).

### Immunization with PfrCelTOS impairs sporozoite infection *in vivo*.

**FIG 1:**
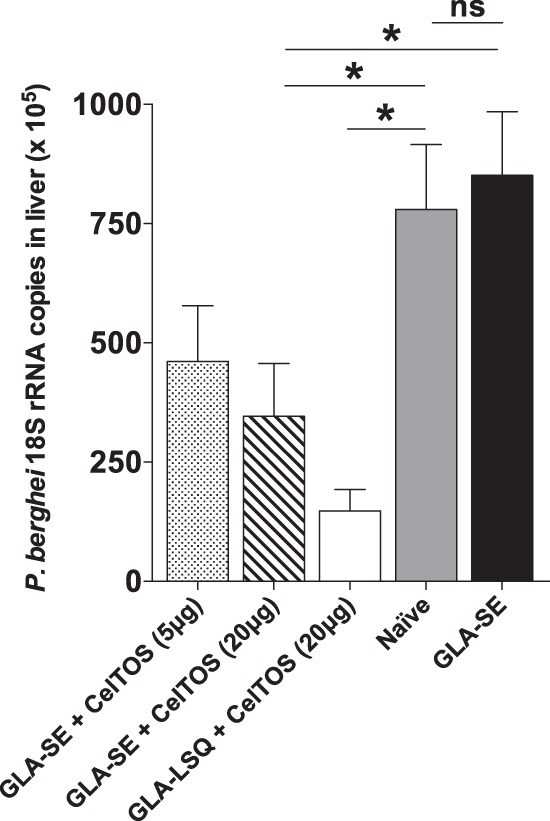
Immunization with PfrCelTOS inhibits sporozoite hepatocyte infection. Immunizations with 20 μg PfrCelTOS in combination with GLA-SE or GLA-LSQ conferred significant protection against challenge with chimeric PbANKA-PfCelTOS(r)_PbCelTOS_CelTOS parasites. Mice immunized with 5 μg PfrCelTOS and GLA-SE had only a modest reduction of liver parasite burden (46% inhibition compared to the GLA-SE control group). The graph represents the results of one experiment. Values are means ± standard errors of the means (SEM) (*n* = 5 mice per group; *, *P* < 0.05; ns, not significant).

In experiments to evaluate the protective effect of immune responses elicited by PfrCelTOS, we found that mice immunized with 20 μg of PfrCelTOS in combination with the glucopyranosyl lipid adjuvant-stable emulsion (GLA-SE) or glucopyranosyl lipid adjuvant-liposome-QS21 (GLA-LSQ) adjuvant system had a significantly reduced parasite liver burden after challenge against chimeric PbANKA-PfCelTOS(r)_PbCelTOS_CelTOS parasites. Mice immunized with 20 μg PfrCelTOS and GLA-SE had a 60% reduction of liver parasite burden compared to that in mice immunized with GLA-SE only (Fig. 1). In addition, animals immunized with 20 μg PfrCelTOS and GLA-LSQ had a 77% reduction of liver parasite load compared to that in naive controls after challenge with PbANKA-PfCelTOS(r)_PbCelTOS_CelTOS parasites (Fig. 1).

### Sera raised against PfrCelTOS recognize P. falciparum sporozoites.

**FIG 2:**
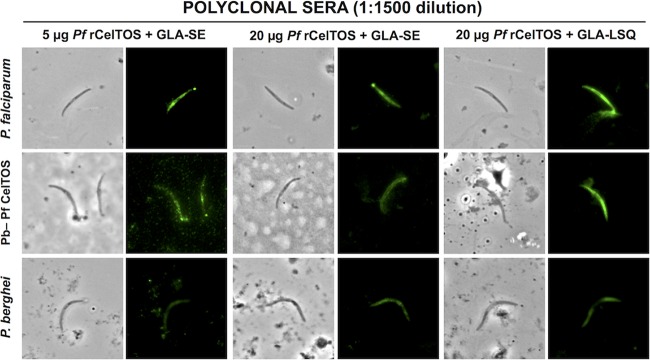
Polyclonal sera and monoclonal antibodies bind CelTOS on sporozoites. P. falciparum (3D7), chimeric PbANKA-PfCelTOS(r)_PbCelTOS_CelTOS parasites, and P. berghei (ANKA) sporozoites were stained with polyclonal sera.

We performed immunofluorescence assays (IFAs) to determine the reactivity of polyclonal sera generated in mice immunized with PfrCelTOS. We showed that the vaccine-induced antibodies are capable of recognizing P. falciparum 3D7 and chimeric PbANKA-PfCelTOS(r)_PbCelTOS_CelTOS parasites. Sera from mice immunized with 5 μg or 20 μg of PfrCelTOS in combination with GLA-SE yielded a fluorescent signal at up to a 1:1,500 dilution when tested against both parasite lines. IFA titers of polyclonal sera generated in mice immunized with 20 μg of PfrCelTOS and GLA-LSQ reached 1:4,500 against the PbANKA-PfCelTOS(r)_PbCelTOS_CelTOS parasites and 1:13,500 against P. falciparum sporozoites. We also found that the anti-CelTOS polyclonal sera were able to recognize WT P. berghei sporozoites, with titers comparable to those reached with PbANKA-PfCelTOS(r)_PbCelTOS_CelTOS parasites (Fig. 2). Preimmune sera or sera from mice injected with adjuvants only did not recognize sporozoites by IFA (see Fig. S4 in the supplemental material).

### Passive transfer of MAb 4D10 partially inhibits sporozoite hepatocyte infection.

In view of the results obtained after immunizations with the PfrCelTOS, we developed monoclonal antibodies (MAbs) 3C3 and 4D10, which are specific for the P. falciparum CelTOS sequences CESQSMNKIGDDLAE and RGNNGHNSSSSLYNC, respectively. Both MAb 3C3 and MAb 4D10 reacted weakly against P. falciparum and PbANKA-PfCelTOS(r)_PbCelTOS_CelTOS sporozoites (data not shown). No cross-reactivity was observed against WT P. berghei. To determine whether these antibodies were capable of neutralizing sporozoite hepatocyte infection *in vivo*, we passively transferred 300 μg of each antibody into naive mice immediately prior to challenge with chimeric PbANKA-PfCelTOS(r)_PbCelTOS_CelTOS parasites. Passive transfer of MAb 4D10 modestly reduced sporozoite infectivity up to 48% compared to that in naive controls. However, the parasite burden in mice receiving MAb 3C3 was not significantly different from that of naive controls that did not receive antibodies but were injected with parasites (see Fig. S5A in the supplemental material). Mice receiving control antibodies that do not bind to sporozoites had a parasite burden comparable to that observed in naive mice (see Fig. S5B in the supplemental material).

### Anti-CelTOS monoclonal antibodies bind to P. falciparum ookinetes and inhibit oocyst formation.

**FIG 3:**
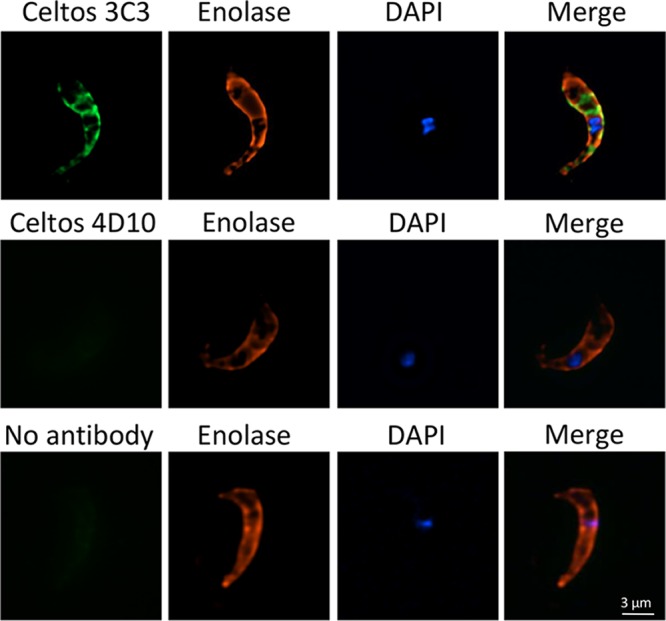
Monoclonal antibody 3C3 binds to P. falciparum ookinetes. Ookinetes were isolated from mosquito midguts 24 h after feeding on an infectious blood meal. Ookinetes were incubated with either MAb 3C3 or MAb 4D10. Anti-P. falciparum enolase antibody (34) was used as a positive control.

**FIG 4:**
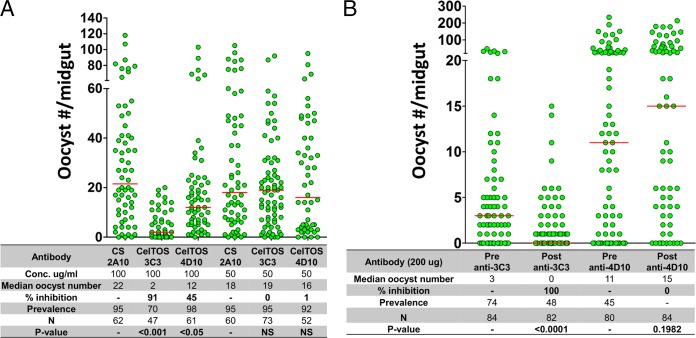
Inhibition of P. falciparum oocyst formation by monoclonal antibodies. The effect MAbs 3C3 and 4D10 on P. falciparum (A) and PbANKA-PfCelTOS(r)_PbCelTOS_CelTOS (B) oocyst formation was determined by SMFAs and PAFAs, respectively. (A) Increasing concentrations of the antibodies were added to P. falciparum gametocyte cultures and fed to A. gambiae female mosquitoes. Control groups of mosquitoes were fed with cultures to which the anti-P. falciparum CSP monoclonal antibody 2A10 was added (35). (B) A. gambiae female mosquitoes (preantibody) were fed on mice infected with PbANKA-PfCelTOS(r)PbCelTOSCelTOS parasites. After feeding, mice were injected intravenously with 200 μg of either MAb 3C3 or MAb 4D10. A second group of mosquitoes (postantibody) were fed on the antibody-treated mice. Percent inhibition is defined as [(control median oocyst number − experimental median oocyst number)/control median oocyst number] × 100. Prevalence, percentage of infected mosquitoes. N, number of mosquitoes analyzed. Horizontal bars represent the median number of oocysts from two independent experiments for results shown in panels A and B. Statistical significance was determined using one-way ANOVA with the Bonferroni multiple-comparison test (A) or the Mann-Whitney U test (B).

Given the critical role of CelTOS for ookinete development, we sought to determine if monoclonal antibodies targeting this protein bind to P. falciparum ookinetes and inhibit oocyst formation in A. gambiae mosquitoes. Immunofluorescence assays showed that MAb 3C3, but not MAb 4D10, clearly binds to ookinetes isolated from mosquito midguts (Fig. 3). This is in contrast to the weak fluorescence observed with sporozoites. In a standard membrane feeding assay (SMFA), P. falciparum gametocyte cultures were mixed with different concentrations of MAb 3C3, MAb 4D10, or control antibody 2A10 and then fed to A. gambiae mosquitoes. Eight days after feeding, we determined the median oocyst number, calculated as described in Materials and Methods. We found that in mosquitoes fed with gametocyte cultures incubated with 0.1 mg/ml of irrelevant antibody (2A10), the median oocyst number was 22. In contrast, in mosquitoes fed with gametocyte cultures incubated with 0.1 mg/ml of MAb 3C3, the median oocyst number was reduced to 2; i.e., there was a 91% inhibition. The same concentration of MAb 4D10 inhibited only 45% of developing oocysts. No inhibitory effect was observed when the monoclonal antibodies were mixed with gametocytes at 0.05 mg/ml (Fig. 4A).

In addition, passive-administration feeding assays (PAFAs) were performed to determine the effect of monoclonal antibodies on oocyst formation by PbANKA-PfCelTOS(r)_PbCelTOS_CelTOS chimeric parasites. Oocyst numbers were determined in A. gambiae mosquitoes fed on mice infected with chimeric parasites, before and after passive administration of either MAb 3C3 or MAb 4D10 (Fig. 4B). The median oocyst number obtained in mice before transfer of MAb 3C3 was 3, and this was reduced to 0 after antibody transfer. In contrast, transfer of MAb 4D10 did not reduce oocyst formation. These results are in agreement with our previous results showing that MAb 3C3 binds to P. falciparum ookinetes and inhibits oocyst formation (Fig. 3 and 4A).

## DISCUSSION

In this study, we report the protective effect of functional immune responses and antibodies against the P. falciparum CelTOS protein. To evaluate *in vivo* responses against this protein, a new chimeric P. berghei parasite line expressing the P. falciparum CelTOS was generated. Chimeric rodent parasites expressing orthologous proteins from human malaria species are practical tools to evaluate immune responses elicited by vaccination or natural infection *in vivo* (14–19). The newly generated PbANKA-PfCelTOS(r)_PbCelTOS_CelTOS chimeric parasite develops normally throughout blood and mosquito stages, and its infectivity is comparable to that of WT P. berghei sporozoites.

Importantly, we used an expression system that has shown to robustly produce recombinant full-length malaria antigen in a conformationally accurate and biologically active state (20). Further, this protein was produced without the use of expression tags. Although tags are useful research tools, they may potentially impact activity and are not appropriate for inclusion in a final vaccine product.

Immune responses were induced by immunization with P. falciparum rCelTOS in combination with the adjuvant systems GLA-SE or GLA-LSQ. This recombinant protein was produced in P. fluorescens, a standardized platform that has been previously used for generating recombinant P. falciparum CSP (20). Mice immunized with 5 μg or 20 μg of PfrCelTOS in combination with GLA-SE or GLA-LSQ mounted immune responses that inhibited infection of chimeric PbANKA-PfCelTOS(r)_PbCelTOS_CelTOS sporozoites. The induced antibodies bind to P. falciparum sporozoites, PfCelTOS in chimeric sporozoites, and WT P. berghei as determined by IFA. The cross-reactivity between PfCelTOS and PbCelTOS in WT P. berghei parasites is consistent with previous studies indicating cross-reactivity between CelTOS expressed in P. berghei and P. falciparum (11), which is likely due to the significant similarities between the amino acid sequences of the P. falciparum and P. berghei CelTOS proteins.

To address the protective and transmission-blocking capacity of antibodies against CelTOS, the MAbs 3C3 and 4D10 were developed. In passive-transfer experiments, we demonstrate that MAb 4D10 can partially inhibit hepatocyte infection by chimeric PbANKA-PfCelTOS(r)_PbCelTOS_CelTOS parasites, while MAb 3C3 appears to have no discernible effect on neutralizing sporozoite infection. In contrast, in SMFA experiments, we found that MAb 3C3 is capable of strongly inhibiting P. falciparum oocyst development in A. gambiae mosquitoes. MAb 4D10 was also capable of significantly inhibiting oocyst development, but its effect was not as strong as that observed with MAb 3C3. In support of these data, MAb 3C3 also inhibited oocyst formation of PfCelTOS(r)_PbCelTOS_CelTOS chimeric parasites. The fact that MAb 3C3 had a stronger oocyst-inhibitory effect than MAb 4D10 may suggest that MAb 3C3's epitope is more available for antibody targeting during the early developmental stages of the parasite within mosquitoes. However, in contrast to the results obtained with monoclonal antibodies, we did not detect an inhibitory effect of anti-CelTOS polyclonal sera on P. falciparum oocyst formation (not shown). It is also worth mentioning that another group mapped a CelTOS peptide (amino acids [aa] 21 to 40) that overlaps with our MAb 4D10 peptide (aa 26 to 40) (21) and found that this peptide bound to liver-derived cell lines (HeLa and HepG2). Therefore, it may have been predicted that MAb 4D10 would be protective at the liver stage and that this region may not have been critical for the oocyst stage. Further studies will be necessary to determine if CelTOS undergoes conformational changes throughout the parasite's life cycle and if certain motifs of this protein become available for antibody targeting at different developmental stages.

We have shown that *in vivo* immune responses and monoclonal antibodies against the P. falciparum CelTOS can inhibit sporozoite hepatocyte infection and impair the parasite's development in mosquito vectors. Our results add to the evidence supporting the protective effect of immune responses against this protein and provide further proof of its potential as a target for vaccine development (7, 11, 22, 23). Additionally, the current study shows for the first time that antibodies against CelTOS not only inhibit sporozoite infection but also inhibit parasite development in mosquitoes. While the anti-CelTOS antibody responses may not be sufficient to confer sterile immunity, our findings should encourage further research aimed at generating a multiantigenic vaccine that could include CelTOS to potentially target the malaria parasite at different developmental stages.

## MATERIALS AND METHODS

### Recombinant CelTOS protein.

Recombinant P. falciparum CelTOS (PfrCelTOS) was produced in a Pseudomonas fluorescens platform using Pfenex Expression Technology (Pfenex, Inc., San Diego, CA) (24) Briefly, the P. falciparum 3D7 (isolate BAD97684) mature CelTOS coding sequence, corresponding to UniProt Q53UB8 amino acids 25 to 182, was optimized for expression in P. fluorescens (DNA2.0, Menlo Park, CA) and cloned into 20 expression plasmids, each with a different P. fluorescens periplasmic secretion leader/ribosome binding site combination. The CelTOS-encoding sequence was cloned in frame with the secretion leader-coding sequence in each plasmid and transformed into 50 P. fluorescens host strains. The resulting expression strains were then screened for protein yield and quality by SDS-capillary gel electrophoresis (CGE) and intact mass analysis, respectively.

A subset of the tested strains was advanced to the 2-liter bioreactor scale for the production of material for preclinical testing. Cells were lysed by microfluidization using 2 passes at 15,000 lb/in^2^ (Microfluidics M-110Y high-pressure pneumatic homogenizer) and loaded into a TMAE HiCap (EMD) anion-exchange capture column, followed by polishing steps using hydroxyapatite (HA) (type I, 40 μm; Bio-Rad) mixed-mode chromatography and MonoQ (GE) strong anion-exchange chromatography. Endotoxin analyses were performed using the Limulus amebocyte lysate (LAL) Endosafe portable test system (PTS) (Charles River Laboratories) following manufacturer-supplied operating procedures. Protein samples were processed prior to intact mass analysis by liquid chromatography coupled to mass spectrometry (LC-MS) to detect any clipped species.

### Antibody development.

The monoclonal antibodies (MAbs) 3C3 and 4D10 were generated by Precision Antibody, a division of A&G Pharmaceutical Inc. (Columbia, MD), using proprietary technology and mouse immunization protocols. MAb 3C3 was generated through immunizations with the CelTOS-derived synthetic peptide CESQSMNKIGDDLAE, whereas peptide RGNNGHNSSSSLYNC was used to generate MAb 4D10. Supernatants from the generated hybridomas were screened by ELISA using PfrCelTOS (Pfenex Inc.) and by Western blotting using a cell lysate from P. fluorescens CelTOS expression strains.

### Generation of DNA constructs and genotyping of the chimeric parasites.

To generate the chimeric parasites where the P. berghei celtos coding sequence (CDS) (Pb*celtos*) (PBANKA_1432300) has been replaced by the P. falciparum *celtos* CDS (Pf*celtos*) (PF3D7_1216600), we used a 2-step gene insertion/marker out (GIMO) transfection protocol (25, 26). In the first step, we deleted the Pb*celtos* CDS and replaced it with the positive-negative selectable marker, to create a P. berghei *celtos* deletion GIMO line (PbANKA-CelTOS GIMO). In order to do this, we generated the pL1960 construct, which is based on the standard GIMO DNA construct pL0034 (25). This construct contains the positive-negative (h*dhfr*::y*fcu*) selection marker (SM) cassette, and was used to insert both the Pb*celtos* 5′ and 3′ gene-targeting regions (TR), encompassing the full-length promoter and transcription terminator sequences, respectively. The linear pL1960 DNA construct was introduced into PbGFP-Luc_con_ parasites (676m1cl1) using standard methods of transfection (27). Transfected parasites were selected in mice by applying positive selection by providing pyrimethamine in the drinking water (27). Transfected parasites were cloned by limiting dilution (28), resulting in the PbANKA-CelTOS GIMO line (line 2217). Correct deletion of the Pb*celtos* CDS was confirmed by diagnostic PCR analysis on genomic DNA (gDNA) and Southern analysis of pulsed-field gel-separated chromosomes as described previously (29). Primers used for PCR genotyping are listed in Tables S1 and S2 in the supplemental material.

In the second step, we replaced the positive-negative SM in the PbANKA-CelTOS GIMO genome with the Pf*celtos* CDS by GIMO transfection to create the P. berghei chimeric CelTOS replacement line. This was achieved by modifying the construct used in the first step (pL1960); specifically, the h*dfhr*::y*fcu* SM cassette was removed and replaced with Pf*celtos* CDS sequence, generating plasmid pL1971. The Pf*celtos* CDS was amplified from genomic DNA of the NF54 strain of P. falciparum. The pL1971 construct was sequenced to ensure that there were no mutations in the Pf*celtos* CDS. The construct was linearized using ApaI and NotI restriction enzymes outside the 5′ and 3′ TRs before transfection. The construct was used to transfect parasites of the PbANKA-CELTOS GIMO line (line 2217cl1) using standard methods of GIMO transfection (25). Transfected parasites were selected in mice by applying negative selection by providing 5-fluorocytosine (5-FC) in the drinking water of mice (30). Negative selection results in selection of chimeric parasites where the h*dhfr*::y*fcu* SM in the *celtos* locus of the PbANKA-CelTOS GIMO line is replaced by the CDS of Pfceltos. Selected chimeric parasites were cloned by limiting dilution (28). Correct integration of the constructs into the genomes of chimeric parasites was analyzed by diagnostic PCR analysis on gDNA and Southern analysis of pulsed-field gel-separated chromosomes as described previously (29). Primers used for PCR genotyping are listed in Tables S1 and S2. This method creates chimeric “gene replacement” P. berghei parasites that lack the Pb*celtos* CDS but express PfCelTOS [PbANKA-PFCelTOS(r)_PbCelTOS_; line 2258cl2] under the control of the Pb*celtos* regulatory sequences.

### Phenotyping of chimeric parasites.

Multiplication of blood stages in mice was determined during the cloning period as described previously (29, 31). Feeding of A. stephensi mosquitoes, determination of oocyst production, and sporozoite collection were performed as described previously (31). Expression of PfCelTOS in chimeric sporozoites was analyzed by immunofluorescence assay (IFA) using sera from mice immunized with PfCelTOS or PbCelTOS (diluted 1:100) (13). Purified sporozoites were fixed with 4% paraformaldehyde in phosphate-buffered saline (PBS) for 20 min on ice and then washed three times with PBS and blocked with 20 μl 10% fetal bovine serum (FCS) plus 1% bovine serum albumin (BSA) in PBS for 30 min at room temperature. The excess blocking medium was removed, followed by the addition of 20 to 25 μl primary monoclonal antibody in 10% FCS plus 1% BSA in PBS (blocking medium) for 1 to 2 h at room temperature or overnight at 4°C. After incubation, the primary antibody was removed and the slides washed three times with PBS, followed by staining with the secondary antibody (Alexa Fluor 488 goat anti-mouse IgG from Life Technologies [catalog number A-11001]) diluted 1:800 in blocking medium for 1 h at room temperature. After washing three times with PBS, nuclei were stained with 2% Hoechst 33342 (Cell Signaling Technology, catalog number 4082S) in PBS for 10 min at room temperature, washed twice with PBS, and left to air dry; this was followed by addition of fluorescence mounting medium (Dako, code S3023). Coverslips were mounted onto the slides, and the slides were sealed with nail polish and left to dry overnight in the dark. The parasites in both blue and green channels were analyzed using a DMI-300B Leica fluorescence microscope and images processed using ImageJ software. The infectivity of chimeric sporozoites was determined by assessing the prepatent period in mice infected with chimeric sporozoites, which was comparable to that observed in mice infected with WT P. berghei sporozoites (see Fig. S1 and S2 in the supplemental material). These results demonstrate that chimeric P. berghei sporozoites expressing PfCelTOS instead of PbCelTOS are fully infectious and able to complete liver stage development in mice.

### Immunizations and sporozoite challenge.

C57BL/6 mice were immunized with 5 μg or 20 μg of PfrCelTOS in combination with 5 μg of the adjuvant systems glucopyranosyl lipid adjuvant-stable emulsion (GLA-SE) and GLA-liposome-QS21 formulation (GLA-LSQ) (IDRI). Intramuscular immunizations were administered in the hind legs on study days 0, 14, and 35. Three weeks after the last immunization, mice were challenged with 5 × 10^3^ sporozoites of the chimeric parasite line PbANKA-PfCelTOS(r)_PbCelTOS_ (2258 cl2) delivered by intravenous (i.v.) tail vein injection.

### IFAs.

Immunofluorescence assays (IFAs) were performed using air-dried sporozoites or ookinetes obtained from mosquito midguts dissected 24 h after feeding on an infectious blood meal. Briefly, a sporozoite suspension (4 × 10^5^ to 6 × 10^5^ sporozoites/ml) or ookinete suspension was air dried at room temperature on poly-l-lysine-coated slides (Tekdon Inc., Myakka City, FL). Parasites were fixed for 30 min using a 2% (wt/vol) paraformaldehyde (Sigma, St. Louis, MO)–1× phosphate-buffered saline (PBS) solution and washed 2 times. Sporozoites were then permeabilized for 30 min using a 0.1% (vol/vol) Triton (Roche, Mannheim, Germany)–1× PBS solution and washed. Antibody or serum samples diluted in 1% (wt/vol) bovine serum albumin (BSA) (Sigma, St. Louis, MO)–1× PBS (1% BSA–PBS) were incubated for 30 min at room temperature. Slides were then washed with 1% BSA–PBS and incubated for 30 min at room temperature with a secondary-antibody solution [Alexa Fluor 488 F(ab′)_2_ fragment of goat anti-mouse IgG(H+L); 2 mg/ml; (Invitrogen)]. Fluorescent sporozoites and ookinetes were visualized under an upright fluorescence microscope (Nikon Eclipse 90i).

### Sporozoite challenge upon passive transfer of monoclonal antibodies.

Three hundred micrograms of the monoclonal antibody MAb 3C3 or MAb 4D10 was passively transferred into naive mice by i.v. tail vein injection 5 to 10 min prior to challenge with 2 × 10^3^ chimeric PbANKA-PfCelTOS(r)_PbCelTOS_CelTOS sporozoites, delivered via the same route. Forty hours later, mice were euthanized and livers harvested to quantify the parasite load by reverse transcription-quantitative PCR (RT-qPCR) (32).

### P. falciparum SMFA.

For the standard membrane feeding assay (SMFA), P. falciparum gametocyte cultures were diluted to 0.03% gametocytemia. Monoclonal antibodies were added to these cultures at a final concentration of 50 μg/ml or 100 μg/ml, and then the cultures were fed to A. gambiae female mosquitoes. Mosquitoes were kept at 27°C in a humidified chamber. Mosquito midguts were dissected 8 days after feeding, and the oocyst number per midgut was determined after staining in a 0.2% (wt/vol) mercury dibromofluorescein salt–1× PBS solution. Percent inhibition was defined as [(control median oocyst number − experimental median oocyst number)/control median oocyst number] × 100.

### P. berghei PAFA.

Passive-administration feeding assays (PAFAs) were performed as previously described (33). Briefly, a group of A. gambiae mosquitoes (preantibody) were fed for 15 min on a mouse infected with PbANKA-PfCelTOS(r)_PbCelTOS_CelTOS parasites. The mouse was then injected i.v. with 200 μg of either MAb 3C3 or MAb 4D10. After 15 min of recovery to allow for antibody dissemination in the bloodstream, the mouse was used to feed a second group of A. gambiae mosquitoes (postantibody). Mosquito midguts were dissected 10 days after feeding, and the oocyst number per midgut was determined after staining in a 0.2% (wt/vol) mercury dibromofluorescein salt–1× PBS solution. Percent inhibition was defined as [(control median oocyst number − experimental median oocyst number)/control median oocyst number] × 100.

### Mice.

Five- to 8-week-old female C57BL/6 mice were purchased from NCI (Frederick, MD). All experimental procedures involving mice were approved by the Institutional Animal Care and Use Committee of The Johns Hopkins University.

### Data analysis.

Statistical analysis comparing means was performed by a two-tailed unpaired Student *t* test with Welch's correction, unless otherwise stated in a figure legend or table. As stated in the legends of the figures, the statistical significance was determined using one-way analysis of variance (ANOVA) with Bonferroni multiple-comparison tests or Mann-Whitney or Mantel-Cox analysis.

## Supplementary Material

Supplemental material
