# Supplementary material for: The Plasmodium falciparum Cell-Traversal Protein for Ookinetes and Sporozoites as a Candidate for Preerythrocytic and Transmission-Blocking Vaccines
Source: Infect Immun. 2017 Jan 26;85(2):e00498-16. doi: 10.1128/IAI.00498-16 (PMC5278177; doi:10.1128/IAI.00498-16)
Supplement: Supplemental material [file supp_85_2_e00498-16__index.html]

The Plasmodium falciparum Cell-Traversal Protein for Ookinetes and Sporozoites as a Candidate for Preerythrocytic and Transmission-Blocking Vaccines — Supplemental material 

# The Plasmodium falciparum Cell-Traversal Protein for Ookinetes and Sporozoites as a Candidate for Preerythrocytic and Transmission-Blocking Vaccines

## Supplemental material

- Supplemental file 1 -

  Fig. S1. Genotype and phenotype analyses of the chimeric *P. berghei* parasite line expressing *P. falciparum* CelTOS. Fig. S2. Liver infectivity of chimeric sporozoites. Fig. S3. Strategy to generate a chimeric *P. berghei* parasite line expressing *P. falciparum* CelTOS. Fig. S4. Preimmune sera and sera from mice injected with adjuvants only do not bind to sporozoites from *P. falciparum* (3D7), chimeric PbANKA-PfCelTOS(r)PbCelTOSCelTOS parasites, or *P. berghei* (ANKA). Fig. S5. Challenge of mice with chimeric PbANKA-PfCelTOS(r)PbCelTOSCelTOS sporozoites after passive transfer of anti-CelTOS monoclonal antibodies. Table S1. Primers for generation of DNA construct. Table S2. Primers for genotyping the chimeric parasite line. Table S3. Developmental characteristics and infectivity of PbANKA-PfCelTOS(r)PbCelTOSCelTOS parasites in *A. stephensi* mosquitoes.

  PDF, 458K
