# Supplementary material for: The Plasmodium falciparum Cell-Traversal Protein for Ookinetes and Sporozoites as a Candidate for Preerythrocytic and Transmission-Blocking Vaccines
Source: Infect Immun. 2017 Jan 26;85(2):e00498-16. doi: 10.1128/IAI.00498-16 (PMC5278177; doi:10.1128/IAI.00498-16)
Supplement: Supplemental material [file IAI.00498-16_zii999091959s1.pdf]

Legends Supplementary Figures

Supplementary Figure 1

Genotype and phenotype analyses of the chimeric *P. berghei* parasite line expressing *P. falciparum* CelTOS.

A. Genotype analysis of Replacement Gene [RG] chimeric parasites (PbANKA-PFCelTOS(r)$_{PbCelTOS}$; line 2258cl2) and their intermediate GIMO mother-line line (2217cl1) by Southern analysis of chromosomes (chr.) separated by pulsed-field gel electrophoresis (PFGE) and by diagnostic PCR analysis.

Hybridisation of chr. of line 2217cl1 with the 3′UTR *Pbdhfr/ts* confirms integration of construct pL1960 (FigS1) into the *Pbceltos* gene on chr. 14. In addition, this probe hybridizes to the GFP-Luc reporter-cassette in chr. 3 (Fig. S1) and to the endogenous *Pbdhfr/ts* on Chr. 7. The correct integration of the *PfCelTOS* expression construct (pL1971; Fig. S1) into the GIMO locus was confirmed by showing the removal of the h*dhfr::*y*fcu* selectable marker (SM) cassette in the cloned chimeric parasite line 2258cl2. The southern blot is hybridized with a mixture of two probes: one recognizing h*dhfr* and a control probe recognizing chr-5. As an additional control (ctrl), parasite line 2117cl1 is used with the h*dhfr::*y*fcu* SM integrated into chr-3.

Genotype analysis by diagnostic PCR analysis of the chimeric parasite line 2258cl2 (right panel) confirms correct integration of the *PfCelTOS* expression cassette. Correct integration is shown by the absence of the h*dhfr*::y*fcu* SM and the *Pbceltos* CDS, the presence of the *Pfceltos* CDS, and the correct integration of the construct into the genome both at the 5’ and 3’regions (5’int and 3’int; see Fig. S1 for primer locations). Primers sequences used are shown in Table S1, while the expected PCR product sizes and the primer numbers are listed in the table below the PCR analysis.

B. Immunofluorescence analysis demonstrating *PfCelTOS* antigen expression in sporozoites of the chimeric line 2258cl2. Salivary-gland sporozoites were stained with sera from mice immunized with *PfCelTOS* and *PbCelTOS* antigens (Alexa Fluor 488, green; nuclear staining with Hoechst-33342). As a control, wild-type (WT; line 676m1cl1) *P. berghei* sporozoites were stained with the same sera. Merged images of the different channels are shown for both chimeric and WT *P. berghei* sporozoites.

C. Fitness assessment of sporozoites the chimeric line 2258cl2. Prepatent period in mice after injection of 10^3 sporozoites of line 2258cl2 and of wild-type *P. berghei* parasites. The prepatent (i.e. the time to reach 1% parasitaemia) was similar in mice infected with 2258cl2 and WT sporozoites (Log-rank (Mantel-Cox) Test; P-value 0.174).

Supplementary Figure 2

Liver infectivity of chimeric sporozoites. Comparison of the *in vivo* infectivity of Pb-Pf CelTOS sporozoites and WT *P. berghei* (ANKA) after i.v. injection. Mean ± SEM; n = 4. ns = not significant.

Supplementary Figure 3

Strategy to generate a chimeric *P. berghei* parasite line expressing *P. falciparum* CelTOS.

This chimeric parasite line was generated using GIMO based transfection technology (1, 2). Using this technology we generated a Double-step Replacement (DsR) mutants (3) that resulted in the CDS of *Pbceltos* being replaced with the CDS of the *Pfceltos* in a two-step GIMO-transfection procedure (Fig. S1-2). First, the *Pbceltos* CDS was deleted by replacement with the h*dhfr::*y*fcu* selectable marker cassette (SM). Subsequently, in these *Pbceltos* GIMO-deletion parasite line the *Pfceltos* CDS was inserted into the same locus thereby replacing h*dhfr::*y*fcu* with the *Pfceltos* CDS. These parasites have replaced full-length *Pbceltos* CDS with that of *Pfceltos* and are SM free. In this chimeric parasite line, PbANKA-PfCelTOS(r)$_{\text{PbCelTOS}}$ (line 2258cl2), the *Pfceltos* CDS is under the control of the *Pbceltos* gene promoter (5'UTR) and transcription terminator (3'UTR) regulatory elements.

A. Schematic representation of the transgenic *p230p* locus of the reporter *Pb*ANKA parasite line *PbGFP-Luc*$_{eef1\alpha}$ (676m1cl1), which used to generate the replacement gene [RG] chimeric parasite line (see **B**). This reporter line expresses a fusion protein of GFP and firefly luciferase (LUC-IAV) under the constitutive *Pbeef1a* promoter and is selectable marker (SM) free. The reporter-cassette is integrated into the neutral *p230p* locus in chromosome 3.

B. Schematic representation of the generation of the chimeric line PbANKA-PfCeLTOS(r)$_{\text{PbCelTOS}}$ (line 2258cl2) where the GIMO deletion-construct (construct 1; pL1960) is used to replace the *Pbceltos* coding sequence (CDS) with the positive/negative selectable maker (SM; h*dhfr::*y*fcu*) cassette, resulting in the generation of the *Pbceltos* GIMO line (*Pb*ANKA-*Pb*CelTOS GIMO; line 2217cl1) after positive selection with pyrimethamine. Construct 1 targets the *Pbceltos* gene by double cross-over homologous recombination. **S*tep 2***: The GIMO insertion-construct (construct 2; pL1971) is used to replace the SM in the *Pbceltos* GIMO line with the *Pfceltos* CDS after negative selection using 5-fluorocytosine (5-FC), resulting in the chimeric line PbANKA-PFCelTOS(r)$_{\text{PbCelTOS}}$ (line 2258cl2). Construct 2 integrates by double cross-over homologous recombination using the same targeting regions (TRs) employed in construct 1, resulting in the introduction of the *Pfceltos* CDS under the control of the *Pbceltos* gene promoter and transcriptional terminator sequences and removal of the SM. Black arrows: location of primers used for diagnostic PCR (see Fig. S1).

Supplementary Figure 4

Pre-immune sera and sera from mice injected with adjuvants only do not bind to sporozoites from *P. falciparum* (3D7), chimeric PbANKA-PfCelTOS(r)$_{\text{PbCelTOS}}$CelTOS parasites or *P. berghei* (ANKA).

Supplementary Figure 5

Challenge of mice with chimeric PbANKA-PfCelTOS(r)$_{\text{PbCelTOS}}$CelTOS sporozoites after passive transfer of anti-CelTOS monoclonal antibodies. (A) 300 µg of mAb3C3 or mAb4D10 were inoculated i.v. into C57BL/6 mice immediately prior to injection of 2 x 10^3 chimeric sporozoites. The graphic represents the results of one experiment. Parasite burden was measured by RT-qPCR in livers harvested 40 hours after challenge. Mean ± SEM; n = 5 mice per group *P

< 0.05; ns = not significant. (B) Irrelevant mAbs 300 µg/mouse were evaluated as described above.

Supplementary Figure 1

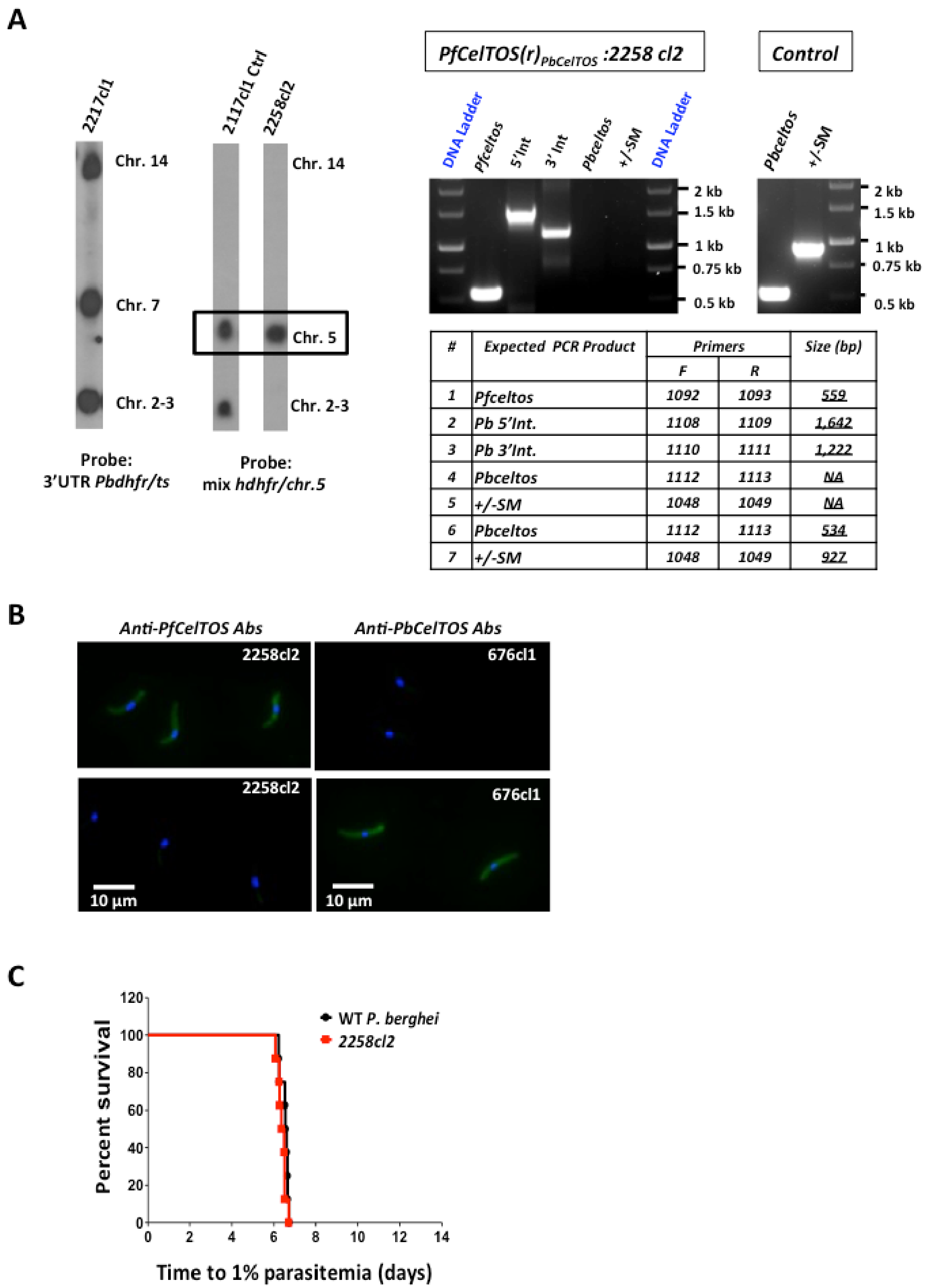

#	Expected PCR Product	Primers		Size (bp)
		F	R	
1	Pfceltos	1092	1093	559
2	Pb 5'Int.	1108	1109	1,642
3	Pb 3'Int.	1110	1111	1,222
4	Pbceltos	1112	1113	NA
5	+/-SM	1048	1049	NA
6	Pbceltos	1112	1113	534
7	+/-SM	1048	1049	927

Supplementary Figure 2

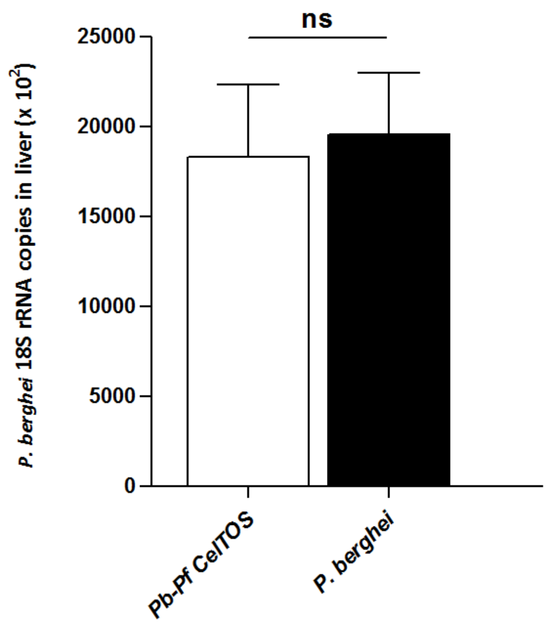

Supplementary Figure 3

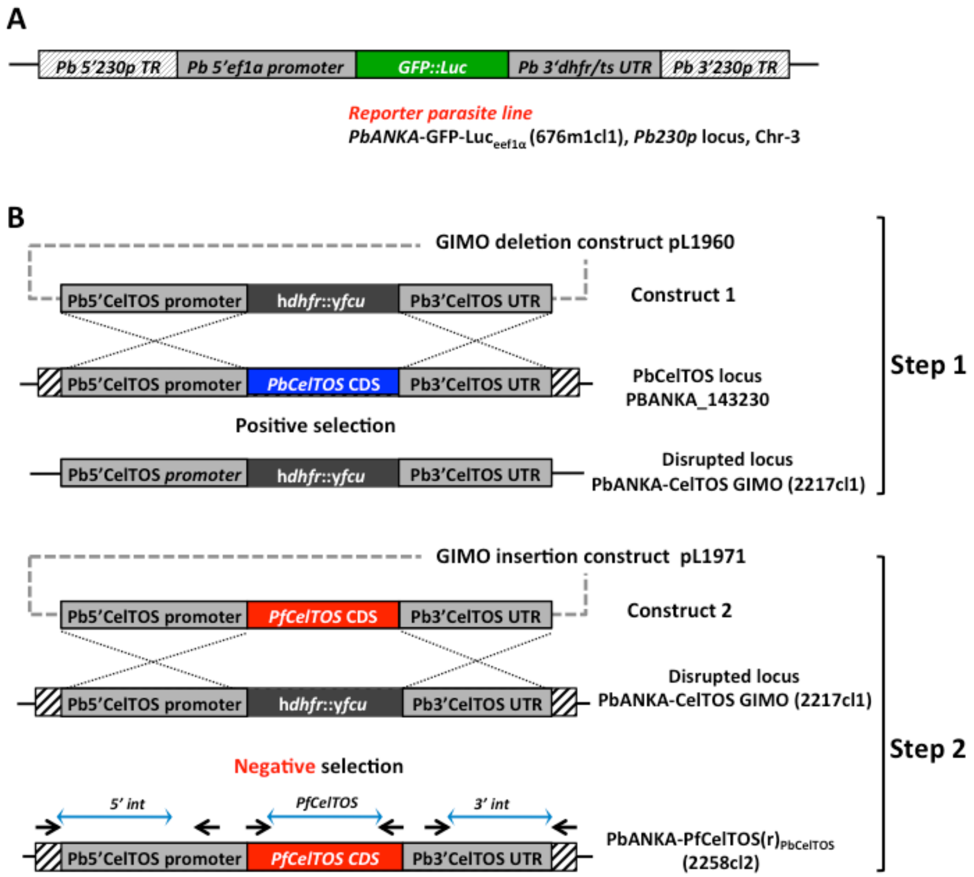

Supplementary Figure 4

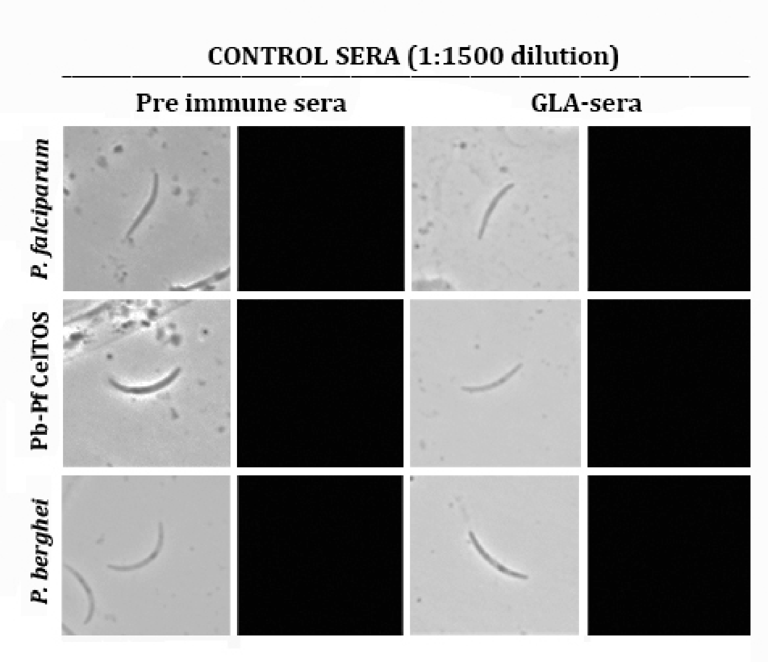

Supplementary Figure 5

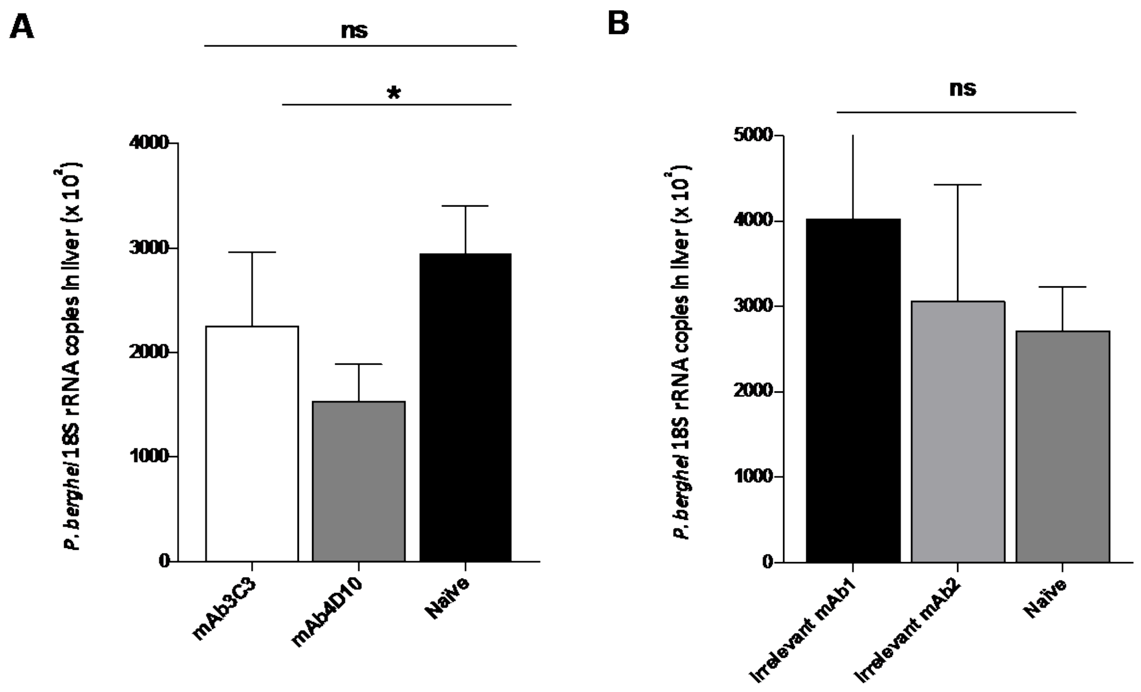

Table S1. Primers for generation of DNA construct

DNA Construct	Primer No.	Primer sequences *	Restriction sites	fragment size (bp)	Description
pL1960	7221	tat**cctgcagg**CTGTAGCATATGTCTATACTAAGC	SbfI	1583	*PbCelTOS* 5'-UTR Promoter sequence, F
	7222	ataagaat**gcggccgc**GTATATTTTAATTATAAATGACAGTTTGAATGATG	NotI		*PbCelTOS* 5'-UTR Promoter sequence, R
	7223	gtgt**caccggcg**CATTTAATATCAATGATAATAATAATAATAATGTAAAC	SgrAI	1003	*PbCelTOS 3'*-UTR sequence, F
	7224	cg**ggatcc**GTATTTTGCACATATCTAATCCGTAAG	BamHI		*PbCelTOS 3'*-UTR sequence, R
pL1971	7191	ataagaat**gcggccgc**ATGAATGCCTTAAGAAGATTACCAG	NotI	587	PfCelTOS, F
	7192	gtgt**caccggcg**CGTGTGATTTTTAATCGAAAAAATCATCTG	SgrAI		PfCelTOS, R

Table S2. Primers for genotyping the chimeric parasite line

Primer No.	Description	Primer sequences *
1048	hDHFR-yFCU (+/-SM) F	ATCATGCAAGACTTTGAAAGTGAC
1049	hDHFR-yFCU (+/-SM) R	CATCGATTCACCAGCTCTGAC
1092	PfCelTOS Integration F	ATGAATGCCTTAAGAAGATTACCAG
1093	PfCelTOS Integration R	CGTGTGATTTTTAATCGAAAAAATCATCTG
1108	PfCelTOS$_{PbCelTOS}$ 5'Int. F	TTAATTTTATCGAATTTGCGCATAACAC
1109	PfCelTOS$_{PbCelTOS}$ 5'Int. R	CTAAGAAAGAGCAAATAACTGGTAATCTTC
1110	PfCelTOS$_{PbCelTOS}$ 3'Int. F	TCTCAGAAAGTGAAGAAAGTTTATCAG
1111	PfCelTOS$_{PbCelTOS}$ 3'Int. R	GATAGCCCTAATGAAAATAATTGCCTC
1112	PbCelTOS F	ACAAAATTGTCAGTTATATCATCTGTCTTTG
1113	PbCelTOS R	ATCATTATCGAAGTTGTCTTCTTCAGTTTC

Table S3. Developmental characteristics and infectivity of PbANKA-PfCelTOS(r)$_{PbCelTOS}$CelTOS parasites in *An. stephensi* mosquitoes[A]

	% Midguts infected	Oocysts/midgut	% Salivary gland infected	Avg. Spz/Salivary gland (x 10^3)	% of infected mice after mosquito bites[B]
Chimeric PbANKA-PfCelTOS(r)$_{PbCelTOS}$CelTOS parasites	75	70	65	10	100%
Pb ANKA	80	65	80	10	100%

[A] Mean of 3 experiments, with at least 20 mosquitoes examined in each experiment.

[B] Five infected mosquitoes were allowed to feed on C57BL/6 mice (n=5) for 5 minutes. Blood stage parasitemia was assessed 5 days later.

Supplementary Bibliography

1. **Lin JW, Annoura T, Sajid M, Chevalley-Maurel S, Ramesar J, Klop O, Franke-Fayard BM, Janse CJ, Khan SM.** 2011. A novel 'gene insertion/marker out' (GIMO) method for transgene expression and gene complementation in rodent malaria parasites. PLoS One **6:**e29289.

2. **Salman AM, Mogollon CM, Lin JW, van Pul FJ, Janse CJ, Khan SM.** 2015. Generation of Transgenic Rodent Malaria Parasites Expressing Human Malaria Parasite Proteins. Methods Mol Biol **1325:**257-286.

3. **Khan SM, Kroeze H, Franke-Fayard B, Janse CJ.** 2013. Standardization in generating and reporting genetically modified rodent malaria parasites: the RMgmDB database. Methods Mol Biol **923:**139-150.
